# Effects of High-Intensity Interval Training in School on the Physical Performance and Health of Children and Adolescents: A Systematic Review with Meta-Analysis

**DOI:** 10.1186/s40798-022-00437-8

**Published:** 2022-04-11

**Authors:** Nikolai Bauer, Billy Sperlich, Hans-Christer Holmberg, Florian A. Engel

**Affiliations:** 1 Institute of Sport and Sport Science, Heidelberg University, Heidelberg, Germany; 2 Working Group Exercise Oncology, Department of Medical Oncology, National Center for Tumor Diseases (NCT), Heidelberg University Hospital, Heidelberg, Germany; 3 Chair of Integrative and Experimental Exercise Science and Training, Institute of Sport Science, Julius-Maximilians-Universität of Würzburg, Judenbühlweg 11, 97082 Würzburg, Germany; 4 Department of Health Sciences, Luleå University of Technology, Luleå, Sweden; 5 Department of Physiology and Pharmacology, Biomedicum C5, Karolinska Institutet, Stockholm, Sweden

**Keywords:** Adolescents, Children, High-intensity interval training, Physical education, Physical fitness, Health-related fitness

## Abstract

**Background:**

Performance of high-intensity interval training (HIIT) by children and adolescents improves physical and health-related fitness, as well as cardiometabolic risk factors.

**Objectives:**

To assess the impact of HIIT performed at school, i.e. both in connection with physical education (intra-PE) and extracurricular sports activities (extra-PE), on the physical fitness and health of children and adolescents.

**Methods:**

PubMed and SPORTDiscus were searched systematically utilizing the following criteria for inclusion: (1) healthy children and adolescents (5–18 years old) of normal weight; (2) HIIT performed intra- and/or extra-PE for at least 5 days at an intensity ≥ 80% of maximal heart rate (HR_max_) or peak oxygen uptake (VO_2peak_) or as Functional HIIT; (3) comparison with a control (HIIT versus alternative interventions); and (4) pre- and post-analysis of parameters related to physical fitness and health. The outcomes with HIIT and the control interventions were compared utilizing Hedges’ g effect size (ES) and associated 95% confidence intervals.

**Results:**

Eleven studies involving 707 participants who performed intra-PE and 388 participants extra-PE HIIT were included. In comparison with the control interventions, intra-PE HIIT improved mean ES for neuromuscular and anaerobic performance (ES jump performance: 5.89 ± 5.67 (range 1.88–9.90); ES number of push-ups: 6.22 (range n.a.); ES number of sit-ups: 2.66 ± 2.02 (range 1.24–4.09)), as well as ES fasting glucose levels (− 2.68 (range n.a.)) more effectively, with large effect sizes. Extra-PE HIIT improved mean ES for neuromuscular and anaerobic performance (ES jump performance: 1.81 (range n.a.); ES number of sit-ups: 2.60 (range n.a.)) to an even greater extent, again with large effect sizes. Neither form of HIIT was more beneficial for parameters related to cardiorespiratory fitness than the control interventions.

**Conclusion:**

Compared to other forms of exercise (e.g. low-to-moderate-intensity running or walking), both intra- and extra-PE HIIT result in greater improvements in neuromuscular and anaerobic performance, as well as in fasting levels of glucose in school children.

## Key Points

- Its favourable cost–benefit ratio makes high-intensity interval training (HIIT) a promising approach to improving the physical health and performance of school children.
- Based on a systematic review with meta-analysis, the impact of HIIT performed as part of physical education (intra-PE) and in other contexts at school (extra-PE) on health-related physical fitness was compared to that of other interventions.
- The 707 school children who performed intra-PE HIIT showed more favourable improvements in neuromuscular and anaerobic performance, as well as in fasting levels of glucose, with large effect sizes.
- The 388 participants in extra-PE HIIT demonstrated even more pronounced improvement in neuromuscular performance, again with large effect sizes.

## Introduction

Most children and adolescents spend a great deal of their time in school and/or with related activities [1]. In general, they do not perform the recommended amount of health-enhancing physical activity [2, 3] and school-based programmes of physical activity can make a significant contribution to maintaining health and preventing disease [4]. In this context, several studies incorporating HIIT (i.e. brief repeated sessions of intense exercise separated by periods of rest or low-intensity exercise [5]) into physical education classes (intra-PE) [6–8] or other activities at school (extra-PE), such as classroom-based exercise [9, 10], have been shown to induce several favourable cardiometabolic [11–13] and neuromuscular adaptations [9, 14–16]; improve parameters related to health and physical fitness in a time-efficient manner [7, 9]; and be experienced as more pleasant than prolonged low-intensity exercise [17–20]. It is necessary to distinguish between intra-PE and extra-PE HIIT, since the duration, specific aims and structure of scheduled physical education and extracurricular sports activities vary. Intra-PE HIIT, which must conform to the school curriculum, appears to be considerably more difficult to implement than extra-PE HIIT.

Indeed, a recent systematic review [21] concluded that HIIT in the school setting is an effective approach for improving parameters of physical fitness related to health in children and adolescents. However, this review emphasized that only 5 of 8 reports documented more pronounced improvements in cardiovascular fitness for those who performed HIIT compared to other forms of exercise (e.g. continuous moderate aerobic running or scheduled PE that included different types of games). Moreover, this review did not distinguish between intra- and extra-PE HIIT and, moreover, unfortunately did not include any meta-analysis designed to quantify the magnitude of the effects observed [21].

Although not focused on school-based activities, the conclusions of a meta-analysis in another systematic review [22] involving 577 children and adolescents (15.5 ± 2.2 years old) were ambiguous as to whether HIIT improves cardiorespiratory fitness to a greater extent than other forms of training. Yet another recent systematic review including meta-analysis [23], involving 563 children and adolescents aged 6–17, but again not based on activities connected with school, found that the somewhat greater improvements achieved with HIIT exhibited only medium effect sizes, with very little heterogeneity across studies. With respect to analogous meta-analyses involving HIIT at school, some have demonstrated significant improvements in cardiorespiratory fitness [10, 24], while others have failed to do so [9, 16].

Thus, to date, no systematic review including meta-analysis has presented a quantitative summary of the effects of intra- and extra-PE HIIT interventions at school. Accordingly, we provide here a comprehensive synthesis with meta-analysis of the effectiveness of intra- and extra-PE HIIT at school on both parameters related to physical fitness and cardiometabolic risk factors in 5–18-year olds.

## Methods

### Literature Searching and Selection of Articles

A systematic review was performed applying the established guidelines of the PRISMA statement [25] and the protocol was registered at PROSPERO (International prospective register of systematic reviews; https://www.crd.york.ac.uk/prospero/). A comprehensive computerized search of the electronic databases PubMed (National Center for Biotechnology Information) and SPORTDiscus (EBSCO) was completed during October and November of 2019 and updated in July of 2021. The electronic databases were scanned with various combinations of the following search strings: “high-intensity interval training” [MeSH terms] or “high-intensity” [All Fields] and “interval” [All Fields] and “training” [All Fields] or “high -intensity interval training” [All Fields] or “high” [All Fields] and “intensity” [All Fields] and “interval” [All Fields] and “training” [All Fields] or “high intensity interval training” [All Fields] or “schools” [All Fields] or “school” [All Fields] or “physical education” [All Fields] or “classroom” [All Fields].

These strings were limited to original research articles published in peer-reviewed journals written in English or German, with no limitation concerning the year of publication. First, the articles identified were entered into EndNote X9 (Clarivate Analytics, Philadelphia, PA, USA), with elimination of duplicates. Thereafter, the titles and abstracts of those of potential relevance were screened with respect to the inclusion and exclusion criteria by NB, with subsequent independent verification by BS. Thereafter, the full texts of the relevant articles were analysed. In addition, the reference lists of the articles thus included were examined manually for additional studies of potential relevance.

### Inclusion Criteria

This systematic review was designed to assess controlled comparisons of HIIT interventions to alternative interventions performed at school and/or to a passive control group. More specifically, the HIIT interventions had to be implemented either in connection with physical education classes (intra-PE) or in other contexts still during school hours (extra-PE, e.g. in the classroom, on school grounds, in-between two classes). In addition, to be considered eligible for inclusion, studies had to fulfil the following criteria:

1. The participants had to be healthy (i.e. free of injuries and chronic or acute diseases), 5–18 years of age, of normal weight, and attending a primary or secondary school.
2. The intra- and extra-PE HIIT interventions had to consist of either (1) 10-s to 5-min intervals of exercise performed at ≥ 80% of maximal oxygen uptake [26, 27], 90–95% of peak heart rate [28], ≥ 100% of maximal aerobic speed (MAS) [29, 30] or with (supra)maximal sprinting [31]; or (2) Functional HIIT (with exercises performed with the participant’s own body weight as resistance, at relatively high velocity, with repetitions and in a fixed, repeated order [9]).
3. The HIIT intervention had to be ≥ 4 weeks in duration or performed in micro-cycles lasting 5–14 days each [32, 33].
4. Parameters related to physical fitness, cardiometabolic risk factors (e.g. diastolic and systolic blood pressure, fasting levels of blood insulin and glucose, and HOMA-IR), and/or physiological performance had to be analysed both prior to and after the interventions.

### Assessment of Methodological Quality

The Physiotherapy Evidence Database (PEDro) scale (described in detail elsewhere [34]) was applied to evaluate the methodological quality of the articles included. In brief, one point was awarded for each criterion fulfilled, with a maximal possible score of 10 points [35, 36]. Tables 1 and 2 document these scores.

**Table 1:** The Physiotherapy Evidence Database (PEDro) scores for the articles included that involved intra-PE HIIT

Publication	Item on the PEDro scale											
	1^a^	2	3	4	5	6	7	8	9	10	11	Total
Baquet et al. [30]	0	0	0	0	0	0	0	1	1	1	1	4
Camacho-Cardenosa et al. [6]	1	1	1	1	0	0	0	1	1	0	1	6
Engel et al. [7]	1	1	1	1	0	0	0	1	1	1	1	7
Martin et al. [8]	1	1	1	1	0	0	0	0	1	1	1	6

^a^Not included in calculation of the total PEDro score

**Table 2:** The Physiotherapy Evidence Database (PEDro) scores for the articles included that involved extra-PE HIIT

Publication	Item on the PEDro scale											
	1^a^	2	3	4	5	6	7	8	9	10	11	Total
Baquet et al. [39]	0	0	0	1	0	0	0	1	1	1	1	5
Baquet et al. [24]	0	1	1	1	0	0	0	0	1	1	1	6
Baquet et al. [41]	0	1	1	1	0	0	0	0	1	1	1	6
Gamelin et al. [40]	1	1	1	1	0	0	0	0	1	1	1	6
McManus et al. [43]	0	1	1	0	0	0	1	0	1	1	1	6
Nourry et al. [42]	1	1	1	1	0	0	0	0	1	1	1	6
van Biljon et al. [10]	1	0	0	0	0	0	0	1	1	1	1	4

^a^Not included in calculation of the total PEDro score

### Data Extraction and Statistical Analysis

Data were extracted from the articles included by NB and the accuracy of this extraction verified by BS. This information concerned publication (e.g. authors, year), characteristics of the study population (e.g. age, sample size), study design (duration of the intervention, number of training sessions per week, type of training), and outcome variables. Here, we only included mean values and measures of variability either obtained from or published elsewhere by the authors themselves.

Pooled standard deviations (SD_pooled_) and standard mean differences (SMD) were calculated. In addition, Hedges’ g effect sizes (ES) and 95% confidence intervals (95% Cl, i.e. the difference between the mean values for the experimental and control interventions, divided by the mean standard deviations for both [37]) were calculated. All calculations were performed with the Microsoft Excel 2016 software (Microsoft, Redmond, WA, USA).

The ES were categorized as follows: < 0.1 trivial; ≥ 0.1 to ≤ 0.3 small; > 0.3 to ≤ 0.5 moderate; and > 0.5 large [38].

## Results

**Fig. 1:**
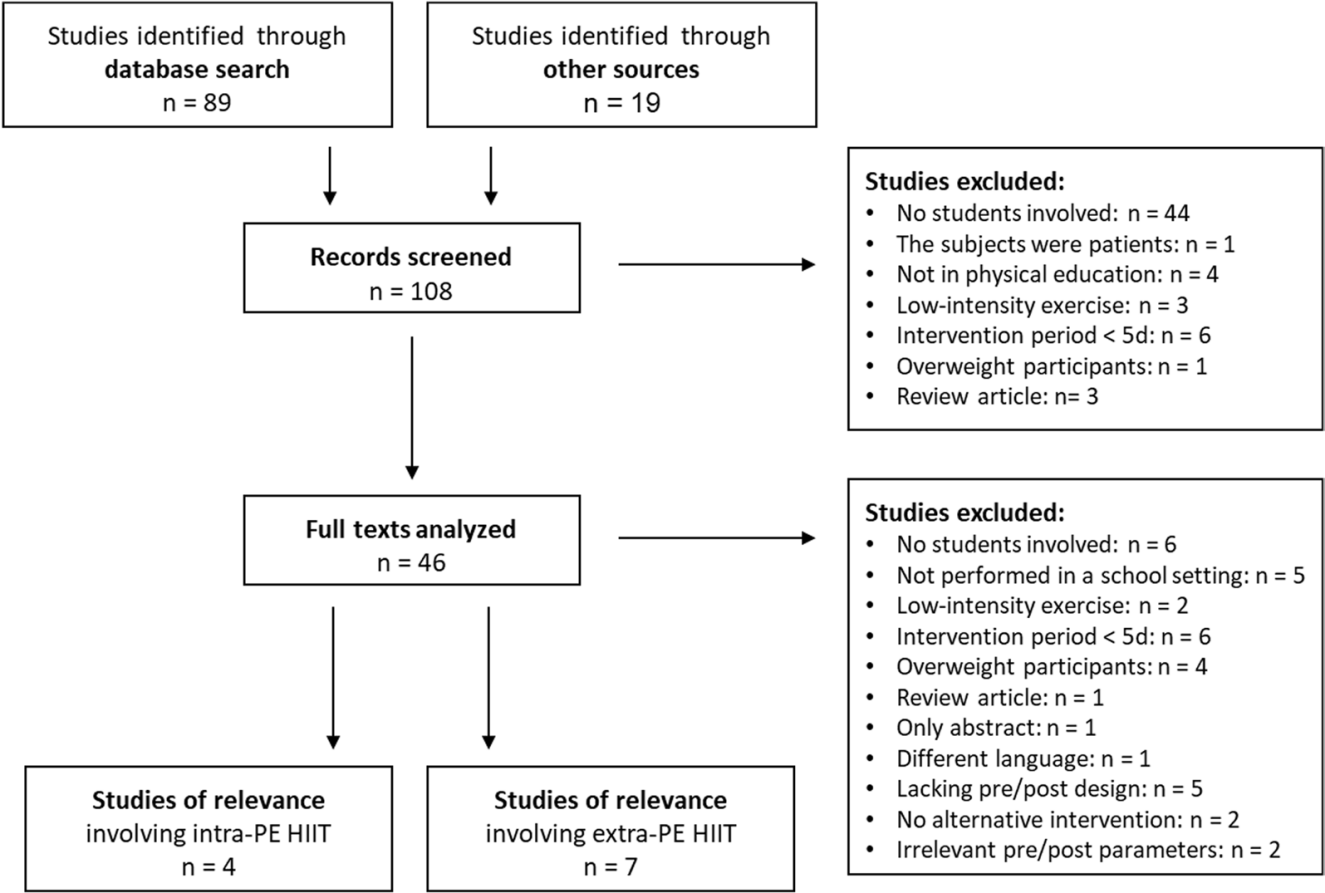
Selection of the studies included

Of the 108 studies initially retrieved, 11 involving a total of 1095 participants were included for analysis (Fig. 1). The characteristics and outcome variables (with statistical analysis) of these 11 studies are summarized in Table 3.

**Table 3:** Characteristics of HIIT interventions performed as a part of physical education (intra) or in another connection (extra) in school

Setting	Study	Intervention	Participants (n)	Age (years)	Type of exercise	Intensity of exercise	Characteristics of the sessions	Duration of study and individual sessions	Frequency of sessions (per week)	Baseline VO_2peak_ [mlmin^−1^·kg^−1^ or l/min^−1^]	%-change VO_2peak_	Outcome: HIIT versus control
Intra-PE	Baquet et al. [30]	HIIE:	503	w = 12.7 m = 12.8	Running + Scheduled PE	100–120% MAS	*n* = 10 W = 10 s R = 10 s W:R = 1 3 sets; 3-min rest	10 wks 25–33 min HIIE	2 with 1 h HIIT and 2 h scheduled PE	n.i	n.i	%BF(m&w) ↑ D7↑ MAS↑ SLJ↑ SHR↔
		CON:	48	w = 13.0 m = 13.5	Scheduled PE	n.i	n.i	10 wks	2	n.i	n.i	
	Camacho et al. [6]	HIIT:	18	11.06	Running (sprints)	89.93% HR_max_ and maximal sprints	*n* = 1 W = 20 s R = 20–60 s W:R = 0.3–1 3–6 sets	8 wks 2.66–8 min	3	46.1 ± 5.7	n.i	FM↔ TFM↔
		CON:	16	11.29	Running	65–75% HR_max_	*n* = 1 W = 2.66–8 min R = 0	8 wks 2.66–8 min	3	45.6 ± 6.6	n.i	
	Engel et al. [7]	HIIT:	39	11.9	Running and game playing	90–100% v_mean_ and all-out sprints	*n* = 4–10 W = 10 s-4 min R = 20 s-3 min W:R = 0.5–1.33	6 wks 20 min	2	n.i	n.i	BMI↑ 6-min Run↑ 20-m Sprint↑ SLJ↑
		GAT:	46	11.4	Running and game playing	65–85% v_mean_	*n* = 1–4 W = 5 min-25 min R = 0–5 min	6 wks 30 min	2	n.i	n.i	
	Martin et al. [8]	HIIT:	18	16.9	Running (sprints)	87% HR_max_ and maximal effort	*n* = 4–6 W = 30 s R = 30 s W:R = 1 4 sets	7 wks 16–24 min	3	47.1 ± 6.3	4.2	VO_2max_↔ FG↔ FINS↑ SRP↔ HOMA-IR↔
		SPE:	19	16.8	Scheduled PE	n.i	n.i	7 wks 60 min	3	46.1 ± 7.3	-6.9	
Extra-PE	Baquet et al. [39]	HIIT:	33	9.5	Running	100–130% MAS	*n* = 5–10 W = 10–20 s R = 10–20 s W:R = 1 1–4 sets; 3 min rest	7 wks 30 min	2	43.9 ± 6,2	8.2	%BF↔ HR_max_↔ MAV↑ VO_2peak_↑
		CON:	20	9.9	Scheduled PE	n.i	n.i	7 wks	n.i	46.2 ± 8.5	-2.0	
	Baquet et al. [41]	HIIE:	36	9.7	Running + Scheduled PE	100–130% MAS	*n* = 5–10 W = 10–20 s R = 10–20 s W:R = 1 1–4 sets; 3-min rest	7 wks 30 min	2 + scheduled PE	n.i	n.i	%BF↔ MAV↑ SLJ↑ SHR↔
		CON:	36	9.7	Scheduled PE	n.i	n.i	7 wks	n.i	n.i	n.i	
	Baquet et al. [24]	HIIT:	22	10.0	Running	100–190% MAS	*n* = 5–10 W = 10–20 s R = 10–20 s W:R = 1 1–4 sets; 3-min rest	7 wks 25–35 min (60 min)	3 + (2 scheduled PE)	51.6 ± 2.8	4.8	BMI↔ HR_max_↔ MAV↑ VO_2peak_↑
		CTG:	22	9.8	Running	80–85% MAS	*n* = 1 W = 6–20 min R = 0 1–4 sets; 5 min rest	7 wks 18–39 min (60 min)	3 + (2 scheduled PE)	50.1 ± 6.0	7.0	
		CON:	19	9.3	Scheduled PE classes	n.i	n.i	7 wks	2	50.6 ± 6.1	-1.8	
	Gamelin et al. [40]	HIIT:	22	9.8	Short intermittent runs	100–190% MAV	*n* = 5–20 W = 5–30 s R = 15–30 s W:R = 0.3–1.5 1–4 sets	7 wks 30 min	3	51.6 ± 2.8	4.8	HR_max_↔ M↑ MAV↑ VO_2peak_↑
		CON:	16	9.3	n.i	n.i	n.i	7 wks 30 min	3	49.9 ± 4.8	-2.4	
	Mc Manus et al. [43]	HIIT:	10	10.4	Cycling	All-out sprints	*n* = 7 W = 30 s R = 2.45 min W:R = 0.18	8 wks 20 min	3	45.5 ± 3.4	11.4	HR_max_↔ VO_2_↑ VO_2peak_↑
		CTG:	10	10.4	Cycling	80–85% HR_max_	*n* = 1 W = 20 min R = 0 min	8 wks 20 min	3	47.0 ± 6.5	7.8	
		CON:	15	10.5	Habitual PA	n.i	n.i	8 wks	n.i	44.7 ± 6.5	1.8	
	Nourry et al. [42]	HIIT:	9	9.7	Running + Scheduled PE	100–130% MAS	*n* = 10 W = 10–20 s R = 10–20 s W:R = 1 4 sets; 3 min	8 wks ca. 30 min	2	37.4 ± 7.3	15.5	%BF↔ FVC↑ HRpeak↔ Ppeak↑ VO2max↑
		CON:	9	10.3	Scheduled PE	n.i	n.i	8 wks	n.i	36.8 ± 7.8	-0.54	
	van Biljon et al. [10]	HIIT	29	11.1	Running/Sprinting	> 80% HR_max_	*n* = 10 W = 60 s R = 75 s W:R = 0.8	5 wks 23 min	3	14.1 ± 7.3	45.4	BMI↔ DBP↓ FG↓ RHR↓ SBP↓ VO2peak↑
		MICT	29	11.1	Walking	65–70% HR_max_	*n* = 1 W = 33 min R = 0	5 wks 33 min	3	11.6 ± 5.1	27.6	
		HIIT & MICT	27	11.1	Running + Walking	65– > 80% HR_max_	*n* = 10 W = 60 s R = 75 s W:R = 0.8 & *n* = 1 W = 33 min *R* = 0	5 wks 23–33 min	3 (9 sessions HIIT/6 sessions MICT)	17.8 ± 9.8	28.1	
		CON	24	11.1	No training/intervention	12.6 ± 6.8	26.19					

^#^: ↑ = significant increase. ↓ = significant decrease. ↔  = no change. %BF = percentage body fat. W:R = work-to-rest ratio. BMI = body mass index. CTG = continuous training group, CON = control group. D7 = maximal distance covered during a 7-min running test, DBP = diastolic blood pressure. FG = fasting level of blood glucose, FINS = fasting level of blood insulin, FM = fat mass, FVC = forced vital capacity. GAT = aerobic endurance training. h = hours, HIIE = high-intensity interval exercises, HIIT = high-intensity interval training, HOMA-IR = homeostatic model of assessment for insulin resistance. HR_max_ = maximal heart rate, HR_peak_ = peak heart rate. M = mass, m = metre, MICT = moderate-intensity continuous training, min = minute, MAS = maximal aerobic speed, MAV = maximal aerobic velocity. n = number, n.i. = not indicated. PA = physical activities, PE = physical education, P_peak_ = peak power. R = rest, RHR = resting heart rate. SLJ = standing long jump, SBP = systolic blood pressure, SHR = 10 × 5 m shuttle run, SPE = standard physical education, SRP = shuttle run performance. TFM = trunk fat mass. v_mean_ = mean running speed, VO_2_ = oxygen uptake, VO_2max_ = maximal oxygen uptake, VO_2peak_ = peak oxygen uptake. W = work

w = women

m = men

### Characteristics of the studies involving Intra-PE HIIT

The studies involving intra-PE HIIT included a total of 707 participants (mean 177 ± 251, range 34–551) with a mean age of 13.2 ± 2.6 years. The HIIT interventions consisted of sessions of running [6, 8], running in combination with game playing [7], or running as part of the scheduled PE [30].

The intervention periods ranged from 6 to 10 (mean 7.8 ± 1.7) weeks, with each individual HIIT session lasting 2.6–33 (mean 18.6 ± 9.8) minutes, excluding warm-up and/or cool-down. Each participant completed two or three HIIT sessions per week, for a total of 11–24 sessions.

The protocols for the sessions of HIIT and rest periods differed. The duration of the HIIT sessions ranged from 10 s to 4 min and the number of repetitions from 1 to 10. The intensity of exercise was 100–120% of MAS [30], 88.5 ± 2.1% (range 87.0–89.9%) of HR_max_ [6, 8] or all-out sprints [6–8]. In one study involving all-out sprints, the mean running speed (V_mean_) was determined employing a 6-min running test [7]. The rest period between sessions of HIIT varied in duration from 10 s to 3 min.

The control interventions consisted of scheduled PE [8, 30], running [6], and running in combination with game playing [7]. The mean length of these interventions was 7.8 ± 1.7 (range 6–10) weeks, with two or three sessions each lasting 2.6–60 min per week, giving a total of 11–24 sessions. The exercise intensities involved were 65–75% of HR_max_ [6] or 65–85% of V_mean_ [7], with two studies failing to specify this intensity [8, 30].

### Characteristics of the studies involving Extra-PE HIIT

The total of 388 (mean and SD 55 ± 30, range 18–109) children and adolescents who participated in this study had a mean age of 10.0 ± 0.6 years. The interventions lasted for 7 ± 1 (range 5–8) weeks and involved running [10, 24, 39, 40], running in combination with scheduled PE [41, 42], and cycling [43]. Each session lasted 20–35 (mean 27.6 ± 4.2) minutes (excluding warm-up and/or cool-down) and each participant performed two or three such sessions per week for a total of 14–24 HIIT sessions per intervention.

Again, the protocols varied. The sessions of HIIT lasted from 5 s to 1 min and the number of repetitions varied from 5 to 20. The intensity of exercise was 100–190% of MAS [24, 39–42], > 80% of HR_max_ [10] or all-out [43]. Rest periods lasted from 10 s to 2.5 min.

The control interventions involved scheduled PE [39, 41, 42], running [24], running in combination with walking [10], and cycling [43], with one study failing to specify the type of exercise [40]. The mean duration was 7 ± 1 (range 5–8) weeks, with individual training sessions lasting 18–39 min. Each participant performed a total of 14–24 sessions of HIIT, i.e. two or three per week. The intensity of exercise was 80–85% of MAS [24] or 65- > 80% [10] or 80–85% [43] of HR_max_, with four studies failing to specify this intensity [39–42].

### Comparison of the Effects of Intra-PE HIIT to Those of the Control Interventions

#### Effects on Parameters Related to Physical Fitness

As shown in Table 4, the outcomes related to physical fitness of intra-PE HIIT that differed from those of the control interventions with a large effect size were as follows: better jump performance (mean *g* = 5.89 ± 5.67; range 1.88–9.90); more push-ups (mean g = 6.22; range n.a.) and sit-ups (mean *g* = 2.66 ± 2.02; range 1.24–4.09); slower maximal aerobic speed (mean *g* = − 1.34; range n.a.); lower maximal oxygen uptake (mean *g* = − 4.51; range n.a.); poorer performance in field running tests (mean *g* = − 2.37 ± 4.64; range − 5.65 to 0.91); and shuttle running (mean *g* = − 16.07; range n.a.). In the case of flexibility, intra-PE HIIT showed a negative effect of moderate size (mean *g* = − 0.45; range n.a.), while for sprint performance the negative effect was small in size (mean *g* = − 0.20; range n.a.).

**Table 4:** Hedges' g effect sizes and 95% confidence intervals for the differences in the effects of intra-PE HIIT and other forms of exercise on parameters related to health

Parameter	Number of studies	Number of participants	References	Hedges’ g	Cl (95%)	
					Lower limit	Upper limit
Body mass index	3	673	[7, 8, 30]	2.76 ± 1.70	2.68	2.84
Weight	3	673	[7, 8, 30]	6.52 ± 10.57	6.27	6.78
Percentage body fat	2	585	[6, 30]	2.91 ± 7.32	2.76	3.06
Jump performance	2	636	[7, 30]	5.89 ± 5.67	5.63	6.15
Performance in field running tests	2	636	[7, 30]	− 2.37 ± 4.64	− 4.06	− 0.68
Number of sit-ups	2	636	[7, 30]	2.66 ± 2.02	2.61	2.72
Fasting level of blood glucose	1	37	[8]	− 2.68	− 2.71	− 2.65
Fasting level of blood insulin	1	37	[8]	11.12	11.1	11.13
Flexibility	1	551	[30]	− 0.45	− 0.48	− 0.42
Homeostatic model of assessment for insulin resistance (HOMA-IR)	1	37	[8]	29.08	29.08	29.08
Maximal aerobic speed	1	551	[30]	− 1.34	− 1.35	− 1.34
Maximal oxygen uptake	1	37	[8]	− 4.51	− 4.88	− 4.15
Number of push-ups	1	85	[7]	6.22	6.15	6.29
Shuttle run performance	1	37	[8]	− 16.07	− 16.43	− 15.71
Sprint performance	1	85	[7]	− 0.20	− 0.2	− 0.19

#### Body Composition

The outcomes related to body composition of intra-PE HIIT that differed from those of the control interventions with a large effect size were as follows (Table 4): a higher percentage body fat (mean *g* = 2.91 ± 7.32; range − 2.26 to 8.08); a higher body mass index (mean *g* = 2.76 ± 1.70; range 0.99–4.39); and greater body weight (mean *g* = 6.52 ± 10.57; range − 2.01 to 18.34).

#### Parameters Related to Metabolism and Blood Pressure

Regarding the parameters related to metabolism and blood pressure, the following effect sizes for the differences between intra-PE HIIT and the control interventions could be estimated (Table 4): a higher fasting level of blood insulin (mean *g* = 11.12; range n.a.) and lower fasting level of blood glucose (mean *g* = − 2.68; range n.a.); and greater HOMA-IR (mean *g* = 29.08; range n.a.).

### Comparison of the effects of Extra-PE HIIT to those of the control interventions

#### Effects on Parameters Related to Physical Fitness.

The outcomes related to physical fitness of extra-PE HIIT that differed from those of the control interventions with a large effect size (Table 5) were as follows: greater flexibility (mean *g* = 3.56; range n.a.); better jump performance (mean *g* = 1.81; range n.a.); higher maximal heart rate (mean *g* = 2.22 ± 2.90; range − 1.01 to 4.8); more sit-ups (mean *g* = 2.60; range n.a.); slower maximal aerobic speed (mean *g* = − 3.96 ± 3.15; range − 8.33 to (−)1.49); lower maximal oxygen uptake (mean *g* = − 1.04 ± 3.27; range − 6.56 to 2.78); and slower maximal rate of work (mean *g* = − 1.19 ± 3.76; range − 3.84 to 1.47).

**Table 5:** Hedges' g effect sizes and 95% confidence intervals for the differences in the effects of extra-PE HIIT and other forms of exercise on parameters related to health

Parameter	Number of studies	Number of participants	References	Hedges’ g	Cl (95%)	
					Lower limit	Upper limit
Weight	6	353	[10, 24, 39–42]	1.49 ± 0.89	0.95	2.04
Maximal oxygen uptake	6	316	[10, 24, 39, 40, 42, 43]	− 1.04 ± 3.27	− 1.45	− 0.62
Maximal heart rate	5	207	[24, 39, 40, 42, 43]	2.22 ± 2.90	1.75	2.69
Maximal aerobic speed	4	226	[24, 39–41]	− 3.96 ± 3.15	− 3.99	− 3.92
Percentage body fat	3	143	[39, 41, 42]	0.52 ± 0.30	− 0.09	1.12
Body mass index	2	172	[10, 24]	0.94 ± 1.58	0.80	1.07
Maximal work rate*	2	53	[42, 43]	− 1.19 ± 3.76	− 3.16	0.79
Blood lactate	1	18	[42]	1.47	1.21	1.74
Diastolic blood pressure	1	109	[10]	0.76	0.54	0.97
Fasting level of blood glucose	1	109	[10]	5.88	5.86	5.89
Fasting level of blood insulin	1	109	[10]	− 0.03	− 0.34	0.28
Flexibility	1	72	[41]	3.56	3.38	3.74
Jump performance	1	72	[41]	1.81	1.24	2.37
Number of sit-ups	1	72	[41]	2.60	2.49	2.72
Systolic blood pressure	1	109	[10]	0.19	− 0.17	0.56

*Maximal work rate (P_peak_), in Watts (W), was determined during an incremental load test on a cycle ergometer [42, 43]

#### Body Composition

The outcomes related to body composition of extra-PE HIIT that differed from those of the other interventions with large effect sizes (Table 5) were as follows: higher percentage body fat (mean *g* = 0.52 ± 0.30; range 0.30–0.86); higher body mass index (mean *g* = 0.94 ± 1.58; range − 0.18 to 2.10); and greater body weight (mean *g* = 1.49 ± 0.89; range 0.38–2.71).

#### Parameters Related to Metabolism and Blood Pressure

The outcomes related to metabolism and blood of extra-PE HIIT that differed from those of the other interventions and their effect sizes (Table 5) were as follows: higher levels of blood lactate (mean *g* = 1.47; range n.a.); higher diastolic blood pressure (mean *g* = 0.76; range n.a.); and higher fasting level of blood glucose (mean *g* = 5.88; range n.a.). In the case of systolic blood pressure, the effect size was small (mean *g* = 0.19; range n.a.) and for fasting levels of blood insulin, trivial (mean *g* = − 0.03; range n.a.).

## Discussion

The aim of this systematic review was to provide a synthetic meta-analysis of the scientific literature regarding the effects of HIIT at school, both during scheduled PE (intra) and in connection with other activities during school hours (extra), on physical fitness and/or individual parameters related to health, as well as to compare these effects to those of other types of exercise.

While previous analyses have assessed the various outcomes of different HIIT protocols in children and adolescents predominantly in the laboratory [44], we focus here on responses to HIIT within the school setting itself. Children and adolescents are sedentary for long periods of time at school, so it seems appropriate to design physical activity with beneficial health effects in this setting [1]. Enhancing physical activities during school hours ensures that more children and adolescents participate [1]. Moreover, the time efficiency of HIIT, especially in school, has been emphasized by several authors [22, 45, 46], indicating the potential of this approach to achieve worthwhile adaptations in relatively short sessions of training [21].

A recent systematic review [21] concluded that HIIT results in reliable and time-efficient improvements in the cardiovascular fitness of children and adolescents. Unfortunately, because of differences in the inclusion criteria and aims, as well as the lack of statistical comparison to control interventions in that earlier work, it is difficult to compare their conclusions directly to those we draw here.

### Effects on Parameters Related to Physical Fitness

The present analysis indicates that both intra- and extra-PE HIIT improve variables related to both neuromuscular and anaerobic performance, including jumping performance and number of sit-ups, in line with previous analyses of different HIIT interventions in young athletes outside of school [44]. In the intra- [6–8, 30] and extra-PE [10, 24, 39–43] HIIT studies examined, two or three sessions of HIIT were performed each week. Moreover, the types of exercise involved in the intra- and extra-PE HIIT interventions were similar. The former involved running [6, 8], running in combination with playing games [7] and running as part of scheduled PE [30], while most of the latter studies involved running [10, 24, 39, 40] or running in combination with scheduled PE [41, 42], with only a single intervention involving cycling [43]. Thus, running was a key component of both intra- and extra-PE HIIT in all cases. The mixture of low- and high-intensity exercise, with frequent accelerations and decelerations and velocities higher than maximal aerobic speed, elicits substantial contribution by anaerobic pathways and neuromuscular load [47], which explains why these effects were more pronounced with HIIT than the other, control types of exercise.

Enhanced cardiorespiratory fitness (CRF) is considered to be a key goal for reducing cardiovascular risk in children and adolescents [48], as well as cardiometabolic risk in adolescents [49]. The indicators of cardiovascular fitness examined here (i.e. maximal aerobic speed, work rate and oxygen uptake, performance in field running tests, shuttle run performance) were all improved to a lesser extent by intra- and extra-PE HIIT than by the control interventions. This is a rather astonishing finding in the light of the fact that in numerous studies, HIIT performed outside the school has led to more improvement in cardiovascular fitness in adolescents [22, 50] and biomarkers of cardiovascular disease in children and adolescents than other forms of exercise [51].

There are several possible explanations for this unexpected result:

1. *The “ActivityStat” hypothesis:* According to this hypothesis, when an individual increases or decreases his/her physical activity in one area, compensatory changes in another area(s) maintain a stable level of physical activity or energy expenditure [52]. This hypothesis is supported by numerous findings that, indeed, during a period when they are participating in an exercise intervention, children reduce their level of other types of physical activity [53–56] ([for an overview see 57]) and/or of exercise during the next day of the intervention [58]. Since not all of the studies analysed here provided detailed information concerning overall physical activity during the intervention period based on objective measurements (e.g. accelerometery), we cannot rule out such compensatory behaviour in connection with the HIIT interventions.
2. *Differences in study design:* We found quite substantial differences between the protocols for the intra- and extra-PE HIIT and the control interventions, especially with regards to the intensity of exercise, as well as the nature and duration of sessions. Comparisons of the effects of exercise intensity often design the control intervention to be similar to the experimental ones with respect to work performed or energy expenditure (i.e. isocaloric comparison) [59, 60]. However, the lack of precise information of this nature in many of the articles analysed here makes it impossible to match these aspects of the different procedures.
3. *Inadequate description of the intensity, duration, and frequency of exercise*: A previous systematic review [61] proposed that an intervention lasting for a minimum of 6 weeks, with 3 or 4 sessions of physical activity per week is necessary to improve CRF in children 6–12 years of age. Here, only two [6, 8] of the four intra-PE HIIT interventions and three [24, 40, 43] of the seven extra-PE HIIT studies fulfilled these criteria. In two [8, 30] of the four intra-PE HIIT investigations, no information was provided concerning the intensity of exercise or number and lengths of the sessions in the control interventions. Of the 7 studies on extra-PE HIIT, six [24, 39–43] lacked any information about exercise intensity or the number, frequency, and duration of control sessions. Furthermore, certain of the reports [8, 24, 30, 39, 41, 42] refer superficially to “scheduled PE”, without providing any specific details.

In fact, more recent studies have confirmed that in children HIIT has less influence on cardiovascular risk factors than other forms of exercise [7, 9].

### Body Composition

The present analysis revealed larger enhancements of body mass index, percentage body fat, and weight following intra- and extra-PE HIIT than after the control interventions (with large effect sizes). Even though exercise elevates energy expenditure, body composition usually only changes in conjunction with a controlled diet ensuring greater energy expenditure than intake [62–64]. A previous meta-analysis of studies conducted outside of school indicated that (1) high-intensity exercise was not more efficient than continuous moderate-intensity training with respect to reducing the weight and percentage body fat of either normal-weight or overweight/obese individuals; and (2) waist circumference and percentage body fat can be reduced by performing HIIT for more than 12 weeks [65].

### Parameters Related to Metabolism and Blood Pressure

The most widely accepted cardiometabolic risk factors in children include long waist circumference, high blood levels of triglycerides, total cholesterol, and high-density lipoprotein cholesterol; increased blood pressure; and elevated fasting levels of blood insulin and glucose [66, 67]. Only two of the articles analysed here [8, 10], involving 146 participants, examined parameters related to metabolic health (i.e. fasting levels of insulin and glucose, as well as HOMA-IR), along with blood pressure, and found that intra-PE HIIT reduced fasting glucose levels (large effect), while extra-PE HIIT reduced fasting levels of blood insulin (trivial effect) to a greater extent than the control interventions. However, this limited amount of data does not allow definitive conclusions to be drawn.

At the same time, a recent systematic review with meta-analysis [68] concluded that a variety of interventions involving exercise of varying intensity at school reduce waist circumference, diastolic blood pressure, and fasting levels of insulin, although these effects were small. In this case, the exercise was performed during or between academic lessons for a period of 8 weeks to 3 years, with a frequency of two sessions per day to one session monthly, with the length of each session ranging from 10 to 150 min.

From a practical point of view, the limitations imposed on intra-PE and extra-PE HIIT by the school curriculum and infrastructure, as well as temporal restrictions, differ. Since our analysis revealed that school children who performed intra-PE and extra-PE HIIT showed improvements in neuromuscular performance, we conclude that both types of exercise induce physiological changes that are beneficial to health.

### Strengths and Limitations of the Present Investigation

1. The major strengths of this review are the extensive statistical analyses performed, including separate analyses of intra- and extra-PE HIIT.
2. As with many meta-analyses, the present one suffers from lack of information concerning the interventions (especially the type, intensity, duration, and frequency of exercise), as well as considerable variation in the protocols involved in the different studies analysed.
3. Depending on the parameter in question, the study populations varied in size from 37 to 673 participants in the case of intra-PE HIIT and from 18 to 353 participants in extra-PE HIIT interventions. Accordingly, the results for certain parameters (e.g. those related to metabolism and blood pressure) should be generalized only with caution.
4. Most of the reports included here do not provide information concerning rates of compliance and adherence, which is essential for evaluating the feasibility of HIIT interventions in school.
5. This analysis does not allow evaluation of potential sex differences in responses to intra- and extra-PE HIIT.
6. In comparisons of the effects of different types of exercise, attempts are often made to ensure that the work performed or energy expenditure (i.e. isocaloric comparison) by the different groups is as similar as possible. However, as mentioned above, the lack of information concerning the type, intensity, duration, and frequency of exercise does not allow the different protocols analysed here to be compared in these respects.

## Conclusions

Based on this synthetic review and meta-analysis, we conclude that children and adolescents who perform intra- and extra-PE HIIT demonstrate more pronounced improvement in parameters related to neuromuscular performance (i.e. number of push-ups and sit-ups), anaerobic performance and flexibility, as well as in fasting levels of blood glucose, than those who engage in other types of exercise. In contrast, intra- and extra-PE HIIT were associated with less improvement in indicators of cardiovascular fitness (maximal oxygen uptake, maximal heart rate, diastolic and systolic blood pressure) and in body composition (body mass index, weight, percentage body fat). In order to be able to draw definitive conclusions, as well as to assess the feasibility of different types of exercise interventions, we strongly recommend that the designs of the HIIT and control interventions be matched appropriately, and that compliance and feasibility be determined. In addition, the methodological quality of future investigations in this area needs to be higher than that of those analysed here.

## Data Availability

The datasets used and/or analysed in the current study will be supplied by the corresponding author upon reasonable request.
